# Value co-creation with family caregivers to people with dementia through a tailor-made mHealth application: a qualitative study

**DOI:** 10.1186/s12913-022-08704-w

**Published:** 2022-11-16

**Authors:** Aber Sharon Kagwa, Hanne Konradsen, Zarina Nahar Kabir

**Affiliations:** 1 Department of Neurobiology, Care Sciences and Society, Division of Nursing, Karolinska Institutet , Alfred Nobels Allé 23, 141 83 Huddinge, Stockholm, Sweden; 2 Department of Gastroenterology, Herlev and Gentofte Hospital, Borgmester Ib Juuls Vej 1, 2730 Herlev, Copenhagen, Denmark; 3 Faculty of Health, University of Copenhagen, Blegdamsvej 3B, 2200 Copenhagen, Denmark

**Keywords:** Value co-creation, Family caregiver, Dementia, eHealth, mHealth, Complex service context, Service dominant-logic, Service marketing, Healthcare

## Abstract

**Background:**

Digitalization has been recognized as an efficient and cost-effective solution to address the increasing need for care due to the ageing population and the rise in people with dementia. This has subsequently increased the need to also care for family caregivers in community settings. Another benefit of digitalization is the introduction of new service concepts within service-dominant logic namely, value co-creation, which is changing the dynamics in healthcare, transitioning from a provider-centric to a customer-centric approach. The literature indicates that this transition is a slow process in healthcare due to the complex service context consisting of multi-stakeholders, with a fragmented decision-making process. This has resulted in limited research on how individuals co-create value through technology. The study aimed to explore how family caregivers to people with dementia living at home, as consumers of healthcare services co-create value in a multi-stakeholder context through a tailormade mHealth application.

**Methods:**

A qualitative explorative design was used. Data were collected through semi-structured interviews with 12 family caregivers of people with dementia living at home. The data were analyzed deductively using qualitative content analysis.

**Results:**

The findings show how family caregivers to people with dementia as healthcare consumers, engaged with a mHealth application and other actors in their service network through different levels of value co-creation activities.

**Conclusion:**

This paper showed a willingness among family caregivers to people with dementia living at home, who mostly consisted of older people, to implement new technology to assist them with their caregiving tasks. The different value co-creation activities adopted by the family caregivers generated different levels of experiential value such as support, knowledge, and increased access to healthcare.

## Background

In recent years, the healthcare sector has begun mirroring the shift from goods-dominant logic to service-dominant logic as seen in marketing and other service fields going from a provider-centric to a customer-centric approach. The facilitation of this shift derives from digitalization and the introduction of concepts like value co-creation. Value in healthcare is multidimensional, subjective, and contextual [1, 2] thus “*value is always uniquely and phenomenologically determined by the beneficiary”* [3] (p. 47). Digitalization has increased customer participation, a key concept in value co-creation, needed for successful health management. To increase participation, service providers must increase autonomy, empower consumers (patients/family caregivers) as decision-making partners, and bridge the knowledge gap during service encounters [2]. Another key aspect is customer resource integration, which is two-fold in the context of value co-creation and managing chronic conditions. The first is *open* where value is integrated through accumulated resources during service encounters. The other is *closed*, which has traditionally been ignored, where the consumer integrates value from their private resources [4].

Eight broad themes of value co-creation activities are presented by McColl-Kennedy et al. [1], regarding the care of people with chronic illnesses consisting of cerebral (thinking) and behavioural (doing) activities in the customer’s value co-creation process. The themes are based on *interaction* defined as the approach taken by customers to engage with individuals in their service network and *activities* defined as cognitive or behavioural approaches to doing things, that can span from simple (low level) to more complex activities (high level). Thereby value co-creation is defined as ‘‘*benefit realized from integration of resources through activities and interactions with collaborators in the customer’s service network*” [1] (p. 375). These value co-creation activities emerged from extensive research on cancer patients as key resource integrators in managing their health, presumed to be transferrable to other healthcare settings regarding care of people with chronic illness: (1) *Cooperating* is a low-level activity, that can include complying with the basic requirements or accepting the information provided by the service provider: (2) *Collating information* includes assorting and sorting information such as management of basic daily activities: (3) *Combining complementary therapies* includes using additional treatment options (e.g., diet, meditation): (4) *Co-learning* includes activities such as actively seeking knowledge outside of the healthcare provider and sharing the acquired information with the healthcare provider: (5) *Changing ways of doing things*, such as partaking in recreational activities and the management of long-term adaptive changes: (6) *Connecting* includes establishing and maintaining relations with individuals from various sources in the service network: (7) *Co-production* includes high level proactive activities such as self-service, assisting in new service development, and reconfiguring one’s medical team: and (8) *Cerebral activities* include self-generated activities like reframing, sense making, positive thinking and emotional labour (e.g., when patients prefer to be alone or don’t share their problems with others) [1].

The need for family caregivers (FCs) to take part in everyday caregiving, continues to increase with the ageing population and the rise of people with dementia (PWD) [5, 6]. Currently, 60% of PWD in Sweden live at home [7], placing a heavy burden on FCs, creating several challenges (i.e., physical, financial, and psychological) causing the experience of caregiver burden [6, 8]. A higher caregiver burden in FCs is associated with a higher intake of prescription drugs and a higher mortality rate compared to non-caregivers. Caregiver burden is also associated with an increased risk of comorbidities such as weight loss, stress, depression, sleep deprivation, physical and mental fatigue, and high blood pressure [9, 10] often leading to institutionalisation [5]. The caregiver burden is reported to be higher among FCs to PWD than in other groups of informal caregivers [8, 11]. These outcomes have significant financial implications, especially in community settings [12] which is increasing the demand for efficient and cost-effective support services [8].

FCs need educational and supportive tools to reduce the negative effects of caregiving [5, 6]. Mobile health (mHealth), healthcare supported by wireless devices, is a cost-effective and efficient tool that facilitates interaction between service providers and healthcare consumers [13], with benefits for FCs including demand-driven, real-time, and time-effective support [12]. Despite this significance, limited uptake of technology is seen among FCs to PWD, who often are spouses and older persons themselves, a user group often overlooked by eHealth developers [8]. Implementation barriers consequently include unsuitable technology and user literacy [13, 14] as well as age [12] and time constraints [6]. The complex service context in which FCs to PWD operate, consists of multi-stakeholders with different roles and needs to fulfil such as the government, patients, FCs, IT developers, and healthcare professionals, creates additional implementation barriers [15]. The cognitive impairment among PWD, often requiring FCs to become their representatives poses another level of complexity [16] making them key organizational resources for service providers [17].

The adoption of value co-creation as a customer-centric approach in healthcare has been slow and continues to result in offerings of products and services being developed without including healthcare consumers [18]. This phenomenon is also identified in eHealth literature where the need for individualised adaptations to facilitate collaborative interactions is gaining recognition [8]. The slow transition has also resulted in shortages of research on how individuals value co-create through technology [18, 19]. Literature regarding FCs and eHealth solutions is not explicitly focused on value co-creation but rather on its practices such as user engagement [5, 6, 20], co-design [21] and improved decision-making and increased empowerment for users [8]. Furthermore, literature regarding healthcare that offers in-depth insight on value co-creation in healthcare settings [1, 2] is based on patients. Value co-creation with FCs is important, especially as the number of PWD continues to increase. This study, therefore, seeks to fill that gap by studying the digitalization of services in a healthcare context and the implication it has for value co-creation at an individual level, through the perspective of FCs to PWD. The aim of the study was to explore how FCs to PWD living at home, as consumers of healthcare services, co-create value in a multi-stakeholder context through a tailormade mHealth application.

## Methods

### Study design

A qualitative explorative design was employed in this study to understand the manifestation of the concept of value co-creation and its underlying processes. The approach is commonly used in qualitative design when investigating new insights and occurrences of a phenomenon that are unknown or little understood [22].

### Setting

The study was conducted in Stockholm city, the capital of Sweden. Qualitative interviews with social care professionals and FCs to PWD generated consumer insight, ideas, and suggestions for a potential mobile application as a tool for support [23]. This contributed to the development of a mHealth prototype named STAV (*STöd till AnhörigVårdare*: Support to family caregivers) [23, 24]. STAV includes the following features: A baseline health self-assessment tool, a chat feature that enables communication between the FC and the social care professional, a digital diary, access to mindfulness sessions, a notification feature where information can be shared, an overview of relevant contact information and web-based links of useful information for the FCs. The intervention in the form of professional support was delivered over eight weeks through STAV, a tailor-made and interactive mobile application to suit the need of the FCs. The support was provided by two members of the research team, both of whom were registered nurses, through the chat feature of the application [24]. STAV was available in both Android and iOS versions.

### Participants

FCs were purposely recruited from the memory clinic of a local hospital. The inclusion criteria of FCs were that they were above the age of 18 years, provided informal care to a person with dementia living at home for a minimum of 6 months, had access to a tablet or a smartphone, had access to broadband/internet at their own expense and could read and write Swedish. Twelve FCs, seven women and five men participated in the interviews. Eleven of them were spouses and one was the daughter of the person with dementia that they cared for.

### Data collection

The interviews were conducted by a research assistant after the eight-week intervention. The interviews were based on a semi-structured interview guide and follow-up questions were used for clarifications purposes. The length of the interviews ranged between 11:42–51:23 min. Two of the interviews were conducted face-to-face in the participants’ own homes. The other 10 interviews were conducted over the telephone due to restrictions related to the COVID-19 pandemic.

### Data analysis

Qualitative content analysis guided the analytic approach [25]. The eight themes of value co-creation activities [1] were used deductively to form a coding scheme through which the interview transcripts were read and reread repeatedly in their entirety. The data was then sorted and condensed within each theme into related value co-creation activities (Table 1). The next step was to derive experiential value from the FCs’ perspectives. To enhance validity, the first and the last authors discussed the findings until a consensus was reached.

**Table 1:** Overview of themes in relation to value co-creation activities and a sample of quotes

Themes	Brief description of the themes	Sample of quotes
**Cooperating**	Includes complying with the basic requirements or accepting the information provided by the service provider.	*“Yes, it was like I felt that after I answered the questions* (the assessment tool), *we are not there yet so I could not contribute anything…” ****FC4.***
**Collating information**	Includes assorting and sorting information such as management of basic daily activities.	*”…the memory is very bad but otherwise, she participates, and she cooks… solves crossword puzzles…. I just try to help with remembering things…” * ***FC11.***
**Combining complementary therapies**	Includes using additional treatment options (e.g., diet, meditation).	*“…the relaxation exercises were very good, when I needed to rest and unwind a little, and just think about myself, then it was great.” * ***FC3.***
**Co-learning**	Includes activities such as actively seeking knowledge outside of the healthcare provider and sharing the acquired information with the healthcare provider.	“*…of course, you get good information, there is a similar application that I looked at, the laws, agreements, and law and such, with information about the disease…” ****FC10.***
**Changing ways of doing things**	Includes activities such as partaking in recreational activities and the management of long-term adaptive changes.	*“…she has had money with her before and she was particular to have her wallet… but it has fallen into oblivion now, but they* (the senior day care*) sometimes have sales there…Maybe they* (the staff) *pay up and then I pay them back …. I have not experienced any problems so far but of course …is a little trickier.” ****FC2.***
**Connecting**	Includes establishing and maintaining relations with individuals from various sources in the service network.	*“My Silvia nurses* (specialized nurses in dementia care) *that I socialise with or that I meet every day…. They are very good to help if you need to discuss. …. they have great contact with us, you can talk about everything possible.” ****FC2.***
**Co-production**	Includes high level proactive activities such as self-service, assisting in new service development, and reconfiguring one’s medical team.	*“He has had a lot of problems with this about the healthcare centre and I have reported it…. It has been a lot of such things.”* ***FC1.*** *“Interviewer: Do you have any suggestions to improve?* *Respondent: Yes, simpler and what is it called, more user-friendly and maybe clearer about this with the chat and how to use it and so on.”* ***FC7.***
**Cerebral activities**	Include self-generated activities like reframing, sense making, positive thinking, and emotional labour (e.g., when patients prefer to be alone or don’t share their problems with others).	“*I think that* (the person with dementia), *we rather keep to ourselves…” ****FC12.***

### Ethics approval and consent to participate

The study was carried out in accordance with the ethical guidelines of the Swedish Ethical Review Authority (Dnr: 2018/5:6). Informed written consent was obtained from all the participants before the interviews. The fact that all the participants could read Swedish and were familiar with using a phone or tablet mitigated the risk of digital vulnerability. The participants were informed that participation in the study was voluntary. All the data were kept confidential, and the participants’ identities could not be connected to the transcribed material and the presentation of the results.

## Results

### Cooperating

The cooperating activities highlighted the complex service context in which FCs to PWD operate. The engagement level with the use of STAV was influenced by the current health condition of the person with dementia. The lowest engagement level was reported by the FCs caring for those in the earlier stages of dementia, who stated that they perceived the application as unsuitable for their current needs. According to the FCs, the caregiver burden was minimal or non-existent at the early stages of dementia which enabled the person with dementia to maintain a lot of their physical and mental capabilities. As described by one of the FCs.

“…*It was too early in his* (the PWD) *disease to get such an access* (the application), *really”****FC3***.

The FCs’ cooperating activities manifested themselves in compliance through direct but seemingly passive interaction with STAV, ranging from daily use to only browsing it a couple of times over the eight-week intervention period. The FCs complied with the basic requirements by downloading the application and replying to the baseline health self-assessment tool and engaging with the provided features. Some FCs also replied to the questions asked by the healthcare professionals through the chat feature, but they did not engage further.

*“Yes, it was like I felt that after I answered the questions* (the assessment tool), *we are not there yet so I could not contribute anything…” ****FC4.***

Cooperating was also related to the FCs’ current state of health or personal life such as illness and stress which reduced engagement with STAV. Some FCs did not engage with certain features such as the mindfulness feature because of a lack of understanding, scepticism, and/or patience.

Cooperating, although considered a low level of value co-creation activity still generated self-reported benefit for the FCs which included increased access to information and healthcare services. These benefits generated knowledge growth, support, and comfort for the FCs. The acquired knowledge about dementia, which was mostly retrieved from the weblinks feature, was factual but general and included information about organisations, disease progression and coping advice. Having all the information organised was time-efficient and therefore highly appreciated. The application’s availability proved to be enough to induce feelings of ease despite the low engagement level. Some FCs described it as a safety net or a friend in need as indicated by one of the FCs.

*“Yes, but it felt good with this application, I did not use it much, but it was there somehow then, and it felt good to have it…” ****FC8***.

### Collating information

The FCs exhibited this level of value co-creation activity by interacting with the healthcare professionals through STAV, particularly its chat feature, asking personalised questions regarding various topics such as progression of the disease and coping skills which required a level of assorting and sorting of information. The main difference in derived value from cooperating activities was the customization of the outcomes to the FCs’ individual needs through real-time and demand-driven interaction with the healthcare professionals. Some FCs also noted that the advice enabled them to do right at once and empowered them in their caregiving role and saved them the effort of having to reach out to traditional healthcare providers.

*”It is an easier way to sign off and then get in touch with someone instead of calling the health service, then you have a small network of contacts there…”* ***FC4.***

The complex service context became evident once again regarding the management of daily activities. The FCs had to balance their activities as well as those of the person with dementia. STAV was utilised as a management tool through the contact- and the diary features that some FCs used to relieve their worries and to track the progression of the disease of the person they cared for. This activity also occurred during the FCs’ closed resource integration where the FCs kept track of and organised the everyday life of the person with dementia they cared for, such as scheduling doctors’ appointments, coordinating with home help service, etc. One FC, for instance, kept track of his spouse’s entire medical history to make each doctor’s appointment efficient. The FCs also assisted the PWD with daily necessities such as medication intake and dressing to more advanced activities such as cooking and doing crosswords as described by one FC caring for his spouse.

*”…the memory is very bad but otherwise, she participates, and she cooks… solves crossword puzzles…. I just try to help with remembering things…”* ***FC11.***

### Combining complementary therapies

This value co-creation activity includes the use of additional support options, such as STAV included mindfulness exercises to be used by the FCs. One FC had taken a previous course in mindfulness while several others experienced meditation through direct interaction with STAV. Although a few FCs found the activity hard to integrate into their daily lives, the overall outcome was positive. The value identified was that the exercises enabled them to de-stress and focus on themselves.

*“…the relaxation exercises were very good, when I needed to rest and unwind a little, and just think about myself, then it was great.”* ***FC3.***

### Co-learning

The findings showed that the FCs exhibited this activity of value co-creation by actively seeking knowledge about dementia through books, newspapers, courses, different websites, and similar applications in addition to using STAV.

“*…of course, you get good information, there is a similar application that I looked at, the laws, agreements, and law and such, with information about the disease…” ****FC10.***

This enabled the FCs to acquire individualised information, improve their knowledge and empower their caregiving role. The acquired knowledge was further integrated as a resource to complement the knowledge obtained through STAV.

A higher level of value co-creation activity was also seen in the utilisation of STAV when the FCs adopted an interactive relationship with the application often using it as a stepping stone to obtain additional information. The weblinks feature for instance was used as a starting point to browse other useful websites. Another FC took matters into her own hands by seeking information using the Google search engine when she did not get a response from the healthcare professional through the application due to technical issues.

### Changing ways of doing things

Activities related to changing ways of doing things took place through the closed resource integration of the FCs. The FCs expressed two such activities, the first being recreation such as walking in nature and going on a vacation with family members. The other is long-term adaptive changes to accommodate the changing needs of their lives in relation to the complexity of caring for a person with dementia including lifestyle changes such as placing the person with dementia in senior day-care, asking other family members for assistance or having to become a financial representative of the person with dementia as described by a FC caring for his spouse.

*“…she has had money with her before and she was particular to have her wallet… but it has fallen into oblivion now, but they* (the senior day care) *sometimes have sales there…Maybe they* (the staff) *pay up and then I pay them back …. I have not experienced any problems so far but of course …is a little trickier.” ****FC2.***

### Connecting

The findings showed that the FCs integrated their resources to co-create value, by connecting with individuals from their entire service network. A connection with individuals using STAV was established through the chat feature, but this connection was not maintained due to the short duration of the pilot study. A long-term connection was therefore mainly established with other individuals from the FCs’ service network. The social care professionals in the municipalities especially provided great support. One FC even considered them as friends to use as sounding boards and socialise with. The support provided by the FCs’ private sources enhanced their well-being through companionship and assistance with everyday care activities.

*”My Silvia nurses* (specialized nurses in dementia care) *that I socialise with or that I meet every day…. They are very good to help if you need to discuss. …. they have great contact with us, you can talk about everything possible.” ****FC2.***

The complex service context also became evident during these value co-creation activities, some FCs expressed the importance of the person with dementia having someone to connect with. One FC saw an improvement in the well-being of the person with dementia after meeting a friend which influenced their caregiving role positively as well.

*”If it has to do with the application itself, it is hard to say, but she feels better and is happier after she met a friend in the building…”* ***FC9.***

### Co-production

The findings showed that the FCs exhibited three co-production activities: (1) reconfiguring healthcare- and social welfare team, (2) redesigning support, and (3) assisting in new service development. Reconfiguring healthcare- and social welfare teams for the FCs related to influencing changes in the public- and market-facing sources of the PWD. Two of the FCs explained how they had managed to stop the relocation of their spouse’s senior day-care centre by contacting local politicians and organising a protest. Another FC acted as her spouse’s representative concerning the medical team by changing the healthcare centre, after experiencing a lot of issues such as the person with dementia did not receive a geriatric doctor after the last one had resigned. The FC further reported these issues to the authorities.

*“He has had a lot of problems with this about the healthcare centre and I have reported it…. It has been a lot of such things.”* ***FC1.***

Redesigning support was exhibited in the findings when the FCs provided feedback to improve STAV. The general feedback was positive. Many thought it was a great initiative that would be used when made available on the market while some gave suggestions for adjustment. One crucial aspect that the FCs discussed was the usability of STAV. The application was easy to use for some FCs while others found it hard to navigate. This outcome was due to a variety of reasons. Some FCs were inexperienced with technology and others stated that they had not been correctly informed on how and when to use the application. For instance, one FC stated that they did not know that the application would be updated or that they were expected to engage with it regularly. The feature of a contact list was not seen by some FCs while others perceived it as an excessive tool in addition to their existing smartphone contact feature. Recurring feedback was to make STAV more user friendly, especially regarding the chat feature. Some FCs said that they disengaged because they did not understand or struggle with the chat. While others found value in the interaction with the healthcare professionals through the application. A few FCs stated that the advice given by the professional was not helpful, while one FC was disappointed because they did not receive a reply to any of her questions due to technical issues. Other suggestions to improve the usability were to use bigger navigation buttons, add more colours, improve the design by enabling the ability to use personalised profile pictures and add notifications to remind the FCs to use the application regularly.

*“Interviewer: Do you have any suggestions to improve?*

*Respondent: Yes, simpler and what is it called, more user-friendly and maybe clearer about this with the chat and how to use it and so on.”* ***FC7.***

The FCs also partook in co-production by assisting with the new service development, where they addressed potential features that would improve STAV. The main feature that the FCs missed was consumer-to-consumer services. The interaction with the healthcare professionals through the application was not enough. Some wanted peer-to-peer support from other FCs in a similar situation through a group chat.

*“…to ask questions and exchange experiences with other people, get to know how they do and how they handle the problems and such and you can share experiences I think would be very good…”* ***FC12.***

On the other hand, a major topic raised was privacy concerns which reduced the engagement level of some FCs with STAV, especially with the chat feature. The FCs wanted reassurance that everything written would remain anonymous. Concerns were especially raised when they saw names “pop up” that they did not recognise.

*“That you have a profile but not that I stand with my full name because I do not want people to be able to Google me and come knocking on the door, maybe then start asking a lot of things or maybe pursue* (the person with dementia).*” ****FC10.***

This made some FCs feel exposed and vulnerable, but others wanted to know the face behind each name. Therefore, a physical meeting before the launch of the application, including a potential group chat feature, was proposed by one FC.

A FC also suggested the ability to post a question or statement anonymously which everyone could read and learn from such as in “frequently asked questions (FAQ)”. Other suggestions were to individualise the information further such as customising the information to their location or providing useful tips about movies and courses.

Finally, the potential target market for STAV was addressed. Some FCs felt like it was well suited for their needs and that of others in a similar situation. Others stated that it was too early and would be more beneficial at a later stage of dementia when the condition had deteriorated. Two of the FCs stated that they would have preferred the support provided through the application right at the beginning of the diagnosis phase to overcome initial challenges.

### Cerebral activities

Reframing and sense-making, emotional labour and positive thinking were the self-generated activities adopted by the FCs through closed resource integration. The reframing and sense-making were mainly in the form of reflecting upon their behaviour as a FC and accepting their position. Some FCs expressed that even though caring for a person with dementia was tough it was something they had learned to manage. Others had intentionally chosen to have a positive outlook on life which subsequently had a positive influence on their caregiving role.

*“No, but you kind of seize the day and live for the moment, anyway it can be much worse…”* ***FC3.***

Emotional labour was expressed by the FCs as feeling alone in their roles as caregivers and described it as a continuous “task” that most in their personal network couldn’t relate to.

*“You are very lonely in this situation. You live with it round the clock…”* ***FC1.***

Some FCs still preferred to keep to themselves, despite the hardship, being reluctant to connect with social care professionals in the municipalities or join family events. Some were also reluctant to ask for help, often driven by the fear of being a burden to others.

*“I think that* (the person with dementia), *we rather keep to ourselves…” ****FC12.***

## Discussion

The study aimed to explore how FCs to PWD living at home, as consumers of healthcare services, co-create value in a multi-stakeholder context through a tailor-made mHealth application. Most research on the resource integration process is based on the patient’s perspective. The current study presents the perspective of not only the FCs but also highlights how they served as a representative of the person they cared for. The study presents new insights on factors that facilitate and challenges of the implementation of value co-creation and digitalization practices from one important user perspective.

### Facilitators of value co-creation and digitalization

A sustainable mHealth implementation and adoption intention among FCs to PWD are reliant on the technology’s perceived usefulness [26, 27]. Generic solutions have proven to create implementation barriers. Thus, it is important to have a clear definition of how and for whom the technology generates value and that the consumers themselves identify the immediate advantage of the technology [26]. The findings of this study showed that the perceived usefulness of the mHealth application amongst most of the FCs increased with the deterioration of the PWD’S condition which resulted in increased caregiver burden. This was highlighted in the value co-creation activities *cooperating* and addressed in *co-production*. This emphasized the need for additional service customization and in-depth market research among this user group which is the strength of the value co-creation process because it facilitates adaptation to suit individuals’ current and future needs through customer-centric approaches [3, 27–29].

Another facilitator of sustainable implementation of digital technology is the FCs’ personal characteristics which include eHealth literacy, motivation, education, and attitudes towards technology [26]. In this study different levels of value co-creation activities were adopted by the FCs, i.e., through open resource integration with the mHealth application and closed resource integration with other actors in their service network which highlighted the complex and multi-stakeholder context in which the FCs operate. Through the value co-creation activity *changing ways of doing things* the FCs engaged in recreational activities to support their well-being and made long-term adaptive changes to adapt to caring for a person with dementia. An added complexity in the FCs’ role as resource integrators was further notable when caring for a person with dementia which was seen, for instance, through the value co-creation activity *collating information.* The FCs not only had to manage their daily activities but that of the PWD as well. This was even reflected in the *co-production activity* when the FCs adopted the role of representatives of the PWD either willingly or out of necessity. Thus, they represented themselves and the PWD in the interaction with their service network and that of the PWD, affirming their role as key organizational actors for the healthcare provider [6, 16, 17].

The unique and contextual resource integration[1, 3, 29] through the different value co-creation activities adopted by the FCs generated different levels of customization and experiential value. One of the main benefits of utilizing the mHealth application reported by the FCs was increased access to healthcare services and information tailored to their individual needs. Benefits such as comfort, support, and knowledge growth, were identified even in the lowest levels of engagement through *cooperating* activities. Another expressed benefit was the reduction of time and effort to search for information suited for this target group [12]. Similar benefits were reported by the FCs displaying a higher level of value co-creation activity such as *collating information*. The main difference was seen in the customization of these outcomes, where the direct interaction with the healthcare professionals especially provided the FCs with real-time, demand-driven and customized support. These benefits empowered the FCs’ decision-making and caregiving capabilities. It was also utilized as a resource by some to address the experience of caregiver burden which had a positive influence on their well-being. The findings, therefore, affirmed the conclusion that mHealth interventions can notably improve the knowledge base of FCs [20], which is a vital aspect in enhancing participation since the accumulated resources enabled FCs to bridge the gap in knowledge on any specific health condition and related services [2]. Being proactive, aside from the interaction with the healthcare provider, through activities such as *co-learning* through a closed resource integration, where the FCs searched for needed information through other means such as books, similar applications, and courses also generated similar results in terms of customized knowledge and support. This further highlights the beneficial influences of the consumers’ characteristics on the implementation of technology and value co-creation practices.

Value co-creation is defined as a collaborative and peer-like process [30]. The mHealth application facilitated the collaboration between the FCs and healthcare professionals through the chat feature. A collaborative relationship was also formed between the FCs and the application itself, by using other features such as the diary feature and the weblink feature which generated feelings of comfort and support. It is, however, important to note that the established relationship between the FCs and the healthcare provider in this testing phase was not maintained due to the short duration of the study period (8 weeks) and the fact that the intervention was carried out by members of the research team. A different outcome could therefore be expected if the intervention was carried out by social care professionals in the municipalities upon the market launch of the mHealth application. This is strongly indicated by the long-term connection established with other actors in the FCs’ service network such as community-based dementia nurses.

### Barriers to value co-creation and digitalization implementation

A barrier for use of digital technology includes a lack of digital literacy [26] which was observed among some of the FCs of the current study. Therefore, ease of installation and ease of use is reported to facilitate technology implementation [31]. On the other hand, ease of use only plays a significant role when the mHealth application itself is perceived as useful [27]. Most of the FCs in this study were spouses of PWD and therefore older persons themselves which resulted in some challenges with digital literacy [32]. Lack of experience with technology is another barrier to access healthcare services [6] which was also observed in the current study. One of the main types of feedback from the FCs was therefore to make the mHealth application more user-friendly which is in line with previous research emphasizing the need for additional education and customizations [8]. Another barrier to digital technology implementation among FCs to PWD includes privacy and ethical concerns such as documenting personal issues [26]. This was observed in the current study indicating that the mHealth application could become a barrier for FCs to PWD to access healthcare services due to privacy concerns and/or potential technical difficulties that may arise. A suggestion brought forth was to have a physical meeting before the launch of a potential mHealth application to put the users at ease.

Creating a superior consumer experience is one of the key roles of service providers in the value co-creation process [2]. This was not always achieved through the mHealth application mainly due to privacy concerns and technical difficulties as previously discussed. The findings also indicate that the healthcare professionals failed to form a partnership with some FCs by not responding to their chat messages and/or not providing adequate explanations regarding the use of the application. This caused some FCs to reduce engagement or completely disengage with the mHealth application. Disengagement also occurred during the FCs’ closed resource integration through self-generated resources such as emotional labour by isolating themselves and not making use of their service network. This further highlighted the complex and fragmented process of value co-creation in healthcare[18] where the consumers’ ongoing participation is greatly influenced by all the actors in the service network as well as their past and present experiences [2].

The literature identifies another barrier for use of digital technology namely workload with multiple commitments such as work, family and caregiving tasks leading to stress and lack of time. This hinders the FCs to PWD to seek adequate information [33] and implement digital technology to support them in their caregiving roles [26]. A few FCs in this study confirmed this by relating workload outcomes such as stress to lowered engagement with the mHealth application. The results reflecting experiences of most of the participants, however, showed the opposite outcome, that the engagement level with the mHealth application increased for those experiencing a higher caregiver burden when caring for a PWD in the later stages of dementia.

## Strength and limitations

The findings of this study present a new perspective in nursing science through the concept of value co-creation which can facilitate the implementation of sustainable mHealth intervention in community settings. The findings of the study may not be transferrable to the broader target market of the mHealth application in other healthcare contexts. Also, it is possible that only those healthcare consumers, i.e., family caregivers, who were digitally literate agreed to participate in the study. The study’s time frame may have created limitations to developing a reciprocal, peer-like relationship with the healthcare provider, which is an integral part of value co-creation. Therefore, future research may examine how the relationship between the healthcare provider and healthcare consumers develops over time through the continuous use of a mHealth application. Furthermore, the perspective of the social care professionals as potential users of the mHealth application was not included in the study, thus leaving out an important user perspective. Lastly, the data collection for this study occurred during the COVID-19 pandemic, which caused the transition from face-to-face interviews to telephone interviews which may have impacted the richness of the data. This might also be the reason why none of the findings extended beyond the eight themes of value co-creation presented by McColl-Kennedy et al. [1].

## Conclusions

This study presented valuable empirical insight into a complex, multi-stakeholder service context by showing how healthcare consumers co-create value through mHealth technology. Furthermore, it suggests that FCs to PWD in community settings, who mostly consisted of older people are willing to integrate digital technology to get support in their caregiving roles. The next step of the current research is to test the effectiveness of the mHealth application in terms of its impact on specific health outcomes.

The findings of the current study can be utilized by researchers, healthcare providers, application developers, and other stakeholders within the service network which is vital for further development and uptake of mHealth services in home-based dementia care. This is needed to address the rising healthcare costs due to the increasing number of PWD and the ageing at home policy adopted in Sweden and other western economies. It is also of great relevance in countries where health- and social care services are not easily accessible.

## Data Availability

The datasets generated during/or analyzed during the current study are available from ZNK upon reasonable request.

## List of abbreviations

FC(s): Family caregiver(s)
PWD: People with dementia
STAV: STöd till AnhörigVårdare:Support to family caregivers
